# Interpersonal Psychotherapy for Eating Disorders

**DOI:** 10.1002/cpp.1780

**Published:** 2012-02-24

**Authors:** Lorna Champion, Michael J Power

**Affiliations:** * University of OxfordOxford, UK

**Keywords:** Eating Disorders, Interpersonal Psychotherapy (IPT), Bulimia Nervosa, Interpersonal Psychotherapy

## Abstract

Interpersonal psychotherapy (IPT) is a leading evidence-based treatment for those eating disorders in which binge eating is a feature. This article begins with a consideration of the rationale for using IPT to treat patients with eating disorders. This is followed by a review of the evidence supporting its use and a brief description of treatment including an illustrative clinical case vignette. The article closes with a discussion of possible future directions for research on IPT for eating disorders. Copyright © 2012 John Wiley & Sons, Ltd.

**Key Practitioner Message:**

- IPT for eating disorders (IPT-ED) closely resembles IPT for depression and primarily focuses on current interpersonal problems. It is well suited for helping patients to address interpersonal difficulties which appear to be maintaining the eating disorder.

## THE RATIONALE FOR USING IPT-ED

There are several reasons to consider in using interpersonal psychotherapy (IPT) to treat patients with eating disorders. The main one is that interpersonal difficulties are common in patients with eating disorders and they appear to contribute to their maintenance. These difficulties may predate the eating disorder or they may have a more recent onset and indeed be a consequence of the disorder. Most adults with eating disorders present in their twenties or early thirties and, on average, have suffered from an unremitting eating disorder for 8 years (Fairburn et al., 2007). As late adolescence and early adulthood are critical periods for the development of relationships, the eating disorder has often resulted in profound interpersonal disturbance by the time that an individual seeks treatment. For example, many patients have had limited experience developing and maintaining intimate relationships, partly as a result of social withdrawal that is a common feature of eating disorders but also due to the low self-esteem which often accompanies the disorder.

These interpersonal difficulties appear to contribute to the maintenance of the eating disorder through a variety of mechanisms. First, patients often become more isolated from the normalizing influence of their peers and, as a result, their psychopathology tends to persist unchallenged. Second, certain eating disorder features may be directly maintained by interpersonal difficulties. For example, both binge eating and dietary restraint tend to occur in the context of, or are exacerbated by, adverse interpersonal events. Third, interpersonal difficulties often serve to worsen self-esteem, which in turn tends to increase patients' efforts to control their eating, shape and weight to feel more in control (Fairburn et al., 2003).

IPT is well suited for helping patients address interpersonal difficulties of this type, thereby removing processes that maintain the eating disorder and facilitating their recovery.

## THE EMPIRICAL STANDING OF IPT-ED

### Bulimia Nervosa

**Figure 1:**
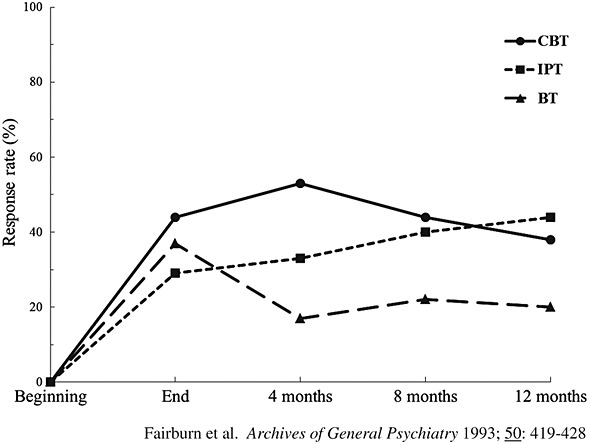
Oxford CBT-IPT-BT study

Two main randomized controlled trials of IPT for bulimia nervosa have been conducted. The first was by Fairburn and colleagues (Fairburn et al., 1991, Fairburn, Jones, Peveler, Hope, & O'Connor, 1993a). Seventy-five patients were randomized to cognitive–behavioural therapy for bulimia nervosa (CBT-BN; Fairburn, Marcus, & Wilson, 1993b), a behavioural version of CBT-BN (behavioural therapy, BT) or IPT. The treatments were carefully monitored for adherence, and at the end of treatment, all patients were entered into a closed 12-month follow-up period. As compared with IPT, CBT-BN was found to be significantly more effective at reducing the key behavioural features of bulimia nervosa at the end of treatment. However, this difference disappeared over the 8 months following treatment due to continued improvement in the IPT group (see Figure 1). The behavioural version of CBT-BN was least effective overall, as a result of substantial post-treatment relapse. Thus, IPT was slower acting than CBT-BN but as effective as in the longer term. Fairburn et al. (1995) found that 6 years after treatment, the majority of patients who had received IPT and CBT-BN had maintained the changes seen at 12 months, and 72% of those who had received IPT no longer met Diagnostic and Statistical Manual of Mental Disorders, 4th edition (DSM-IV) criteria for an eating disorder (Fairburn et al., 1995). IPT and CBT-BN also resulted in an equivalent and lasting decrease in general psychiatric features as well as an improvement in self-esteem and social functioning. The fact that CBT-BN and IPT differed in their temporal effects and were superior to BT indicates that the improvements were not simply the result of non-specific psychotherapeutic processes.

These findings were replicated in a second, much larger (*n* = 220), two-centre study conducted at Stanford and Columbia (Agras, Walsh, Fairburn, Wilson, & Kraemer, 2000). Again, CBT was found to be superior to IPT at the end of treatment, but by 8-to-12-month open follow-up, the two treatments were equivalent. An additional goal of this second study was to identify differential predictors of response to IPT and CBT, the hope being that this would allow patients to be matched to the two treatments. However, no such moderators were identified (Agras et al., 2000).

A further study has been conducted to examine whether those women with bulimia nervosa who do not respond to CBT respond to IPT or antidepressant medication (Mitchell et al., 2002). The findings of this study are difficult to interpret because of a high rate of non-acceptance of the second-line treatment.

### Anorexia Nervosa

Only one study of the use of IPT in the treatment of anorexia nervosa has been conducted (McIntosh et al., 2005). Fifty-six patients were randomized to IPT, CBT or non-specific supportive clinical management. At the end of treatment, IPT was found to be the least effective of the three treatments. However, at long-term follow-up (on average 6.7 years following treatment), no significant differences were found on any outcome measures among the three psychotherapies. Patients in IPT, in particular, appeared to have improved over the follow-up period. The findings of this study should be interpreted with caution, however, given its limitations. In particular, the small sample size and the fact that many of the patients were sub-threshold cases of anorexia nervosa with a weight above the widely used diagnostic cut-off point.

### Eating Disorder Not Otherwise Specified

There have been two main studies of the use of IPT in the treatment of patients with eating disorder not otherwise specified (NOS). These have focused on a subgroup of these patients, namely those with a diagnosis of binge eating disorder who are also overweight.

The first of these studies (Wilfley et al., 2002) replicated the findings of an earlier study (Wilfley et al., 1993) by using a larger sample size (*n* = 162) and tighter controls. Patients were randomized to group CBT or group IPT and then followed-up for 12 months. The patients in the two treatment conditions showed an almost identical, and marked, response over the course of treatment and open follow-up. These findings are suggestive of a non-specific psychotherapeutic response.

In the second more recent trial (Wilson, Wilfley, Agras, & Bryson, 2010), three treatments were compared: IPT on a one-to-one basis, guided cognitive–behavioural self-help (CBTgsh; Fairburn & Carter, 1997) and behavioural weight loss treatment (BWL). Patients (*n* = 205) were randomly assigned to the three psychological interventions. There was no difference between the three groups in terms of their specific eating disorder psychopathology at the end of treatment, a finding consistent with the earlier study. However, at 2-year follow-up, IPT and CBTgsh were found to be significantly more effective than BWL in eliminating binge eating. Further evidence consistent with a specific treatment effect for IPT resulted from the moderator analyses. A high frequency of binge eating at baseline was associated with reduced efficacy of CBTgsh and BWL but not IPT. In addition, at the 2-year follow-up, low self-esteem was associated with a lower remission rates in BWL but not IPT.

No studies have addressed the use of IPT in patients with other forms of eating disorder NOS. This is a significant omission as patients with eating disorder NOS, other than binge eating disorder (BED), are the most common diagnostic group seen in clinical practice and these patients have eating disorders as severe and longstanding as patients with bulimia nervosa (Fairburn et al., 2007). In the absence of evidence to guide the management of these specific patients, the UK National Institute for Health and Clinical Excellence recommended that the clinician follows the treatment guidelines for the eating problem that most closely resembles the clinical presentation of the patient in question (NICE, 2004). This would suggest considering using IPT with those patients with eating disorders in which binge eating is prominent, so long as they are not significantly underweight.

### Conclusion

Four conclusions may be drawn from this body of research:

1. IPT is the leading empirically supported alternative to CBT for bulimia nervosa (NICE, 2004), but it takes longer to achieve its effects. There are no empirical grounds for matching patients to CBT or IPT.
2. IPT cannot be recommended as a treatment for anorexia nervosa.
3. IPT is one of the recommended treatments for BED, the other leading treatments being an adaptation of CBT-BN (Fairburn et al., 1993b), enhanced CBT (Fairburn et al., 2008a) and guided CBTgsh (Grilo, 2006; Fairburn & Carter, 1997).
4. There is a need for research on the use of IPT in patients with eating disorder NOS other than BED.

## THE PRACTICE OF IPT-ED

IPT for patients with bulimia nervosa (IPT-BN; Fairburn, 1997) was derived from IPT for depression (Klerman, Weissman, Rounsaville, & Chevron, 1984) and closely resembles it. IPT-BN has been subsequently modified to create IPT-ED, a version that is designed to be used with any form of eating disorder so long as the patient is not significantly underweight.

IPT-BN and its successor IPT-ED are based upon a somewhat different view of the relationship between the target disorder and interpersonal functioning compared with that held by IPT for depression. This stems from the differing nature of eating disorders and depression. This has resulted in three main adaptations to IPT for depression.

The first two adaptations are related to the chronic, rather than episodic, nature of eating disorders. In our experience, adult patients present for treatment 8 years, on average, after the onset of their disorder. As a result, the patient's current interpersonal difficulties are often different from those which may have operated at the disorder's onset, unlike in the case of depression. Indeed, as discussed earlier, the interpersonal problems detected may be a consequence of the eating disorder rather than being involved in its onset. Therefore, the IPT-ED therapist does not focus on the onset of the disorder but rather on the patient's current interpersonal functioning and circumstances.

Second, a new problem area has been added to address the possible effects of a longstanding eating disorder on an individual's life plans. Late adolescence and early adulthood are the time when most people settle upon their future course in life in terms of their career, lifestyle and place of living. In a subgroup of our patients, this important formative process has been stalled, or may not even have begun, as a result of the eating disorder's pervasive, disruptive influence. As a result, these patients feel ‘at sea’ in terms of their goals and aspirations and this has a marked secondary effect on their relationships and overall functioning. IPT is well suited to addressing this problem which is broader in nature than the types of problem subsumed under the problem area of ‘role transitions’. For this reason, we think it is useful to view ‘life goals’ as a distinct problem area.

The third adaptation concerns the primary mechanism through which we believe that IPT-ED works. IPT-ED helps patients improve their interpersonal life which has a positive effect on the way that they evaluate themselves. This change in their self-evaluation, and the resulting increase in self-esteem, in turn brings about a reduction in the eating disorder features (although it is worth noting that IPT-ED may also have a direct and immediate effect on some reactive features of an eating disorder, such as binge eating). This model of how IPT-ED works remains untested (see Murphy, Cooper, Hollon, & Fairburn, 2009), but it is consistent with its slower mode of action compared with CBT.

Given that IPT-ED is designed to work primarily indirectly, through the patient making significant and long-lasting interpersonal changes, day-to-day fluctuations in the expression of the eating disorder are of less concern than are symptom changes in IPT for depression. As a result, ongoing symptom monitoring, beyond Phase One, is not a feature of IPT-ED. Indeed, it is avoided as in-session attention to eating disorder features can lead patients to engage in such extreme ruminative thinking about shape, weight and eating that it interferes with their ability to think about, and address, their interpersonal problems.

IPT-ED does not give the patient the ‘sick role’ in the same way as IPT for depression. This modification mainly arises from the differing nature of the disorder being treated. More specifically, the patient is encouraged to maintain their occupational and social obligations, where possible, throughout treatment. The patient is seen as responsible for interpersonal change from the outset of treatment. IPT-ED is designed as an outpatient-based treatment. As patients with eating disorders may be at risk of suicidal behaviour and a variety of physical complications, it is necessary at the outset to establish that it is safe and appropriate to provide outpatient treatment. Guidelines for assessing and managing risk, as well as information on the physical effects of eating disorders, are provided by Fairburn, Cooper and Waller (2008b). Clinicians providing IPT-ED need to draw on general knowledge of eating disorders including their key features, co-morbidities and prognosis (Fairburn & Harrison, 2003).

IPT-ED generally involves 16–20 50-minute treatment sessions over about 4–5 months. Like IPT for depression, the treatment has three phases, each with its own aims, strategies and procedures.

*Phase One*—This typically occupies three to four sessions. The first aim of this phase is to engage patients in treatment and describe the rationale and nature of the treatment. The second aim is to jointly identify and agree upon the current interpersonal problem, or problems, which will be the focus of the rest of the treatment.

*Phase Two*—This is the main body of the treatment and comprises up to 14 weekly sessions. The goals are that the patient first understands the nature of the identified interpersonal problem(s) and then addresses it (or them). IPT for depression categorizes interpersonal problems into one of four ‘problem areas’: grief, interpersonal role disputes, role transitions and interpersonal deficits. Both problem area-specific and generic IPT strategies and procedures are used to address these difficulties.

*Phase Three*—This generally occupies the last three sessions. There are two aims: the first being to ensure that the changes made in treatment are maintained and the second being to minimize the risk of relapse in the longer term.

For details about the general practice of IPT, readers should consult the original IPT ‘manual’ (Klerman et al., 1984) or the more recent version of it (Weissman, Markowitz, & Klerman, 2000).

### Phase One

Phase One usually occupies three to four sessions and, as noted above, has two main aims.

1. *Engaging patients in treatment and describing the rationale and nature of IPT-ED*—The therapist explains that to help people break out of a self-perpetuating problem such as an eating disorder, it is necessary to address processes which appear to be contributing to its maintenance. The patient is informed that interpersonal difficulties are common in patients with eating disorders, even though many people have limited awareness of them due to the distracting influence of their preoccupation with thoughts about eating, shape and weight. It is explained that such difficulties appear to contribute to the maintenance of the eating disorder in a variety of ways (as summarized earlier). It is often useful to give patient-specific examples, related to their individual circumstances, of how interpersonal issues may maintain eating disorders. It is made clear that treatment focuses on the patient's current interpersonal difficulties rather than on their eating because the goal is to help patients resolve these difficulties, the expectation being that this will then result in a resolution of their eating problem. In our experience, focusing on the eating problem tends to distract the patient and therapist from this task.

Patients are also forewarned that the treatment changes in style over time. The therapist might say something along these lines:

This initial phase of treatment will involve a review of your past and present relationships, and during this time I will take the lead in asking you questions. This phase of treatment will end with us agreeing upon the problem or problems that should be the focus of the rest of treatment. Thereafter our sessions will change in style. You will become largely responsible for the content of the sessions and I will take more of a back seat role. Gradually we will learn more about your interpersonal difficulties and ways of changing them. Your role will be not only to explore these difficulties in our treatment sessions but also to think about what we have discussed between the sessions. Furthermore, whilst you are having treatment, it is important to experiment with making changes in your life and relationships. Doing so will help us better understand the nature of your problems and may suggest further ways of changing.

It is important that the patient understands that the treatment is time-limited and has a fixed number of sessions. This helps the therapist to stress the need for patients to make treatment a priority in their life.

2) *Identifying current interpersonal problems and choosing which problems should become the focus of treatment*—Three sources of information are used to identify current interpersonal problems:

(i) *A history is taken of the interpersonal context in which the eating problem developed and has evolved*—This highlights links, where available, between changes in the eating problem and interpersonal events and circumstances, thereby stressing the possible importance of interpersonal processes. This helps patients see the relevance of IPT and gives clues as to the sorts of difficulties which may be occurring at present. For most patients, this review should be relatively brief and take only two sessions.
(ii) *An assessment is made of the quality of the patient's current interpersonal functioning and life circumstances*—The therapist conducts a detailed and systematic review of the patient's current social network and current circumstances (termed the ‘interpersonal inventory’). In addition, the therapist asks about significant interpersonal changes over the last few years including interpersonal ‘exits’ and changes in their life circumstances. Enquiry is also made as to whether patients are clear about their goals in life and whether they believe that they are achieving these.
(iii) *The precipitants of changes in eating are identified*—In each of the sessions in Phase One, the therapist also asks whether there have been any changes in the patient's eating since the last session (e.g., intensification of dieting, binge eating and purging) and, if so, enquires about the interpersonal context in which they occurred. Since it is common for changes in eating to be precipitated by interpersonal events, they may serve as ‘markers’ of current interpersonal problems.

By the third or fourth session, the nature of the patient's interpersonal difficulties should be clear. The next step is to decide which problem should become the focus of the remainder of the treatment. This decision should be a joint one. Current interpersonal problems should be chosen rather than those difficulties that have exclusively occurred in the past.

### The Problem Areas

Five types of interpersonal problem tend to be present in patients with eating disorders. These differ somewhat from those identified by Weissman and colleagues in patients with depression. This is not surprising given the differences between the disorders and the patient groups. As noted earlier, patients with eating disorders tend to be younger and suffering from an unremitting, rather than an episodic, disorder.

1. *Lack of intimacy and interpersonal deficits*—In our experience, the most common interpersonal problem encountered in patients with eating disorders is a lack of close or satisfactory intimate relationships, romantic or otherwise. This may be considered a variant of ‘interpersonal deficits’, as recently redefined by Weissman, Markowitz, and Klerman (2007). Whereas some patients describe a scarcity, in general, of interpersonal relationships and feelings of isolation, many, more specifically, lack satisfying intimate relationships. IPT-ED aims to encourage these patients to consider their own expectations and needs in relationships and to take steps to meet these. In doing so, it may be helpful to review the patient's past relationships with the aim of identifying any recurrent problems.
2. *Interpersonal role disputes*—Interpersonal role disputes are also common in these patients. Disputes of this nature may be with any figure of importance in patients' lives, including partners, family members, friends and employers. Such disputes are often the result of each party having differing expectations. The aim of IPT-ED is to help clarify the nature of the dispute, understand the processes through which the dispute is maintained, consider the possibilities for change on both sides (including communicating more effectively and reconsidering expectations) and then to actively explore these. The outcome may be a renegotiation of the relationship or its dissolution.
3. *Role transitions*—Problems with role transitions are seen when the patient has difficulty coping with a major life change that involves a change in role. Such changes are common in this patient group given their age. Frequently encountered examples include moving away from home and establishing independence from parents, starting a first job or having a partner for the first time. However, the difficulties encountered in this area are not confined to the problems of late adolescence and early adulthood. They also include problems coping with other life changes such as changing jobs, getting married and becoming a parent. Patients often need help to understand the notion of ‘role transitions’ and the associated difficulties. The goal of treatment is to help the patient give up the old role and adopt a new one. This involves re-evaluating the old role as well as exploring what the new role involves and how it can be mastered.
4. *(Complicated) Grief*—Problems associated with the death of a loved one are not common in patients with eating disorders given their age. When they do occur, it is important to help patients think, in detail, about the events surrounding the loss and express their feelings about it. Reconstructing the lost relationship, both its positive and negative aspects and the feelings associated with it, is also of importance, as it counters the idealization that commonly occurs. As patients become less focused on the past, they are helped to think about the future and to create new interests and relationships.
5. *Life goals*—As discussed earlier, problems concerning future life plans are present in a subgroup of patients and interfere with their interpersonal functioning. Treatment is an opportunity for such patients to consider their aspirations, to take steps towards meeting them and to make related changes in their life. Typically, it is not addressed in isolation, but only after progress has been made on an associated problem area and patients are beginning to think about their future.

When more than one interpersonal problem is identified, a decision needs to be made about which problem or problems to address. In general, it is only possible to tackle one or two problems during treatment. Our strategy is to address those whose resolution is likely to have the greatest impact on the patient's interpersonal functioning and self-esteem. Clearly, they must also be viewed as a problem by the patient.

### Phase Two

Once the problem area(s) have been agreed by both the patient and the therapist, treatment enters Phase Two. The second and third phases of the treatment are very similar to IPT for depression, as described in the IPT manual, although in IPT-ED, the patient is placed under greater general pressure to change.

The sessions from this point on are largely patient-led. The therapist's stance is largely non-directive and only sparing use is made of more directive strategies (e.g., advice, reassurance), although some psycho-education about the nature of eating disorders is provided. A more directive style is avoided as it can undermine the patient's level of activity in treatment. However, the therapist is active and adopts a strategic approach in sessions, striving to keep the patient focused on the agreed problem area while at the same time considering the treatment's goals. Sessions begin by reminding patients of the session number and how many remain. This is followed by a general enquiry such as ‘How have things been since we last met?’

IPT-ED makes use of a range of therapeutic techniques as employed in IPT for depression (e.g., clarification and exploratory techniques, decision analysis, communication analysis and role play). These techniques are used to support the therapeutic strategies that are specific to the identified interpersonal problem area (as detailed above). In common with IPT for depression, IPT-ED also helps the patient to explore their feelings in relation to interpersonal issues and use these to guide interpersonal change. The initial task in Phase Two is for patients to further characterize the identified problem(s). Generally, after several sessions, the patient has moved on to consider ways of changing. The therapist then helps the patient to think through all the options available (‘decision analysis’), together with their implications, to arrive at a course of action. In general, possible solutions are not offered nor are opinions expressed about what the patient should do. Patients are seen as responsible for reaching their own conclusions about the best course of action. Subsequently, patients' attempts to change become the focus of the sessions.

The need for the patient to change is stressed at regular intervals. It is important to note that this is general pressure to change rather than pressure to take a specific course of action or giving instructions about how to bring about change. Progress in treatment becomes an iterative process. Through making changes, patients gain a greater understanding of their problems and, as a result, are able to make progressively more strategic and influential changes. Furthermore, as patients experience successes resulting from their attempts to change, their confidence about making further changes increases.

As noted earlier, the primary focus of IPT-ED is on current interpersonal problems and not on eating disorder features, which can serve to distract patients and therapists from their main task. Therefore, in Phase Two, changes in the eating disorder are no longer asked about. Interestingly, after a few weeks of treatment, most patients make few, if any, references to their eating disorder. If they do, the therapist shifts the topic on to the interpersonal context of the eating problem (e.g., ‘It sounds as if you have been struggling with your eating this week. What has been happening in your life and relationships?’). Sometimes, under these circumstances, it is helpful to restate the rationale of the treatment as this can help address any concerns patients may have about the absence of a direct focus on the eating disorder.

### Phase Three

Ending the treatment is an important part and an interpersonal event in its own right. The final sessions are held at 2-week intervals, thus allowing patients increase time to continue to make changes on their own with less input from the therapist. There are two related goals. The first is to ensure that the changes that have been made continue following termination, and the second is to minimize the risk of relapse.

While sessions continue in the same style as Phase Two, there is a review of what has been achieved in treatment and consideration of the future. When reviewing progress, the therapist helps patients to project forwards to the future and consider what is likely to happen and how they can continue to build on what they have achieved so far. The therapist also helps patients identify future interpersonal challenges and consider ways in which these might be managed given what has been learned in therapy.

In Phase Three, as well as at intervals earlier in treatment, the interpersonal competence of the patient is highlighted so that patients regard themselves as responsible for change rather than attributing it to the therapist. The therapist notes that, although he or she has helped the patient think through her problems, the patient has actually made the changes that have taken place.

An assessment of the state of the patient's eating problem is also made in Phase Three. Patients are reminded that it often takes a further 4–8 months for the full effects of treatment to be realized and that it would be unwise to seek further treatment during this period. However, we do remind them of the need to continue to address their interpersonal difficulties to make further progress with regards to the eating problem.

It is important to ensure that patients understand that their eating problem is likely to remain an Achilles heel in the sense that a lapse may be experienced in the future, especially at times of interpersonal difficulty. We encourage patients to view any deterioration in the eating problem (e.g., a return of binge eating or strict dieting) as being likely to be a marker of an interpersonal problem and therefore a cue to evaluate what is happening in their life and, perhaps, to take corrective action.

It is unusual for patients receiving IPT-ED to have difficulty accepting the end of treatment. Perhaps, in part, because it is made clear from the outset that treatment has a fixed end. Nevertheless, therapists should always ask patients how they feel about the ending of treatment. It is helpful to acknowledge that patients may feel apprehensive or sad about treatment ending but also to emphasize what has been achieved and to stress the patient's competence at dealing with future areas of difficulty.

A post-treatment review session is provided approximately 20 weeks after the end of treatment. This appointment is designed to let the patient and therapist review interpersonal progress, discuss setbacks and reassess the state of the eating problem.

### Illustrative Case Vignette

The following case history concerns a 32-year-old, married, mother of two who worked as a teacher. Her general practitioner referred her for the treatment of an eating disorder NOS, similar in nature to bulimia nervosa. This was a longstanding problem, which had begun in adolescence.

#### Phase One

Treatment began with the therapist taking a history of the interpersonal context in which the eating problem developed. It emerged that the patient began dieting as a young adolescent and started to binge eat and vomit a year later. During this time, she described feeling alone and isolated. When she started college, the eating problem became more severe. She found her course work to be more difficult than previously. She reported that her response to any life stress was to binge eat and then vomit. In her early twenties, she met her future husband. This was the first time in her life that she was able to get close to someone.

During the first few treatment sessions, the therapist also asked the patient about each occurrence of binge eating and vomiting and the interpersonal context in which they occurred. This revealed that they usually occurred in the evenings, following disagreements with her husband.

In the fourth session, the patient and therapist agreed that treatment should focus on her difficulties with her husband and her desire to spend more time with friends. At the end of this initial phase of treatment, the therapist reminded the patient of the change in session style that was about to take place.

#### Phase Two

The first few sessions of Phase Two were devoted to obtaining a greater understanding of the difficulties that the patient had with her husband. The patient reported that she tended to ‘go along with’ whatever her husband wanted to do but that sometimes she would become ‘fed up’ and shout at him. She appeared very distressed when describing this.

In these initial sessions of Phase Two, the patient expressed concern that her eating had not improved. The therapist reassured the patient that it was likely to take time for her eating to improve and that she should focus instead on thinking about, and making changes to, her life and relationships. From this point onwards, the patient and therapist focused their sessions on the patient's relationship, and the patient did not mention her eating, weight or shape.

To explore the problems with her husband further, the therapist and patient reviewed, in detail, a recent argument that they had experienced. The patient described how she had wanted to spend an evening having dinner out with her friends but that ‘as usual’ her husband had already made plans of his own, meaning that she would need to stay in to look after the children. When the patient's husband arrived home that evening, the patient explained how she could ‘no longer take it’ and shouted at him. This erupted into a lengthy argument. The therapist asked the patient to take her through, as accurately as she could remember, precisely what she had said to her husband. By doing so, it became clear that the patient had not communicated her needs clearly and unambiguously. By reflecting back to the patient what she had said, and more importantly what she had not said, the patient was able to see that perhaps her husband had not realized that she had wanted to go out that evening and was therefore puzzled as to why she was upset when he returned.

Over several sessions, the patient was helped to think through the different options available to deal with similar situations. At first, she struggled with this, as she believed that to have a happy marriage, she had no option but to let her husband do as he wished. She was scared that being open with him about having made plans of her own would result in him leaving her. However, with some encouragement, she was able to talk through each of her options. She became aware that she could carry on doing as she always had done and not tell her husband what was on her mind but she recognized that this would continue to lead to her feeling resentful and to disagreements between them. She considered various alternatives: she could tell him that she already had plans; she could say that she wanted to go out with him; she could discuss the issue more generally with him, or she could hire a babysitter and go out as well. The patient decided that she would like to speak to her husband more generally about her concerns and, in particular, that she would like to have time socializing with her friends.

The patient was somewhat hesitant about implementing this plan as she feared her husband would be upset with her. However, after further considering her husband's likely reaction and practising what she might say (through role playing), she was eventually able to arrange a time to speak to him. She was delighted with the result as the discussion went well and he suggested that, in future, they plan together when they wanted to go out. He also strongly encouraged her to see friends more.

During the following weeks, the patient continued to make further interpersonal changes. She reported that her marriage had improved and she felt closer to her husband. They had started to socialize more as a couple and were better able to resolve disagreements without fighting. Her relationships with her friends developed and she was able to meet them regularly.

#### Phase Three

In the last phase of treatment, the patient's progress in treatment was reviewed. The patient was pleased with the changes that she had made, both in her marriage and in her social life. As a result, she described feeling more self-confident. She was asked to consider possible future interpersonal challenges and the ways in which she might address them, using what she had learned in treatment. She reported that she was more aware of the difficulties in her life and that, instead of avoiding thinking about them; she was able to think about options for resolving them. She envisaged that further difficulties might occur in relationships, e.g., when she took on greater responsibility at work, but that she now felt more able to be assertive about her own needs. She said that she had learnt that not only did this help her to feel more satisfied in relationships but also that the other party was often pleased that she had been clearer and more open about her expectations.

In the penultimate session, the therapist raised the topic of the patient's eating problem. The patient explained that in the initial weeks of treatment, she had been concerned that the eating problem was getting worse but that in the last 2 months or so, she had been binge eating and vomiting much less frequently. She said that she had realized that overcoming the eating problem did not require her to be more disciplined about eating but rather it involved her addressing what was happening in her life. The therapist told her that clinical experience and research evidence led us to expect further improvement over the next 4–8 months if she continued to focus on what she had learned in treatment.

#### Follow-up

The therapist saw the patient for two follow-up sessions, one approximately 20 weeks after treatment had ended and one a further 20 weeks after that. During this time, the patient had continued to work on the changes that she had made during treatment. She described enjoying her relationship with her husband and reported that she now felt able to express her thoughts and feelings to him. Her husband had even remarked to her how nice it was to get to know his wife better. At the same time, she was enjoying spending time with her friends and had organized a book club to help them meet up regularly. She reported that she felt more confident about herself generally as a result of the changes that she had made in her life. Over the same period, her problems with eating had continued to improve and she said that she was no longer ‘obsessed’ with her eating, weight and body shape.

## FUTURE DIRECTIONS

There is a clear need for more research on the use of IPT in the treatment of patients with eating disorders. In bulimia nervosa, the body of evidence supporting its use is far more modest than it is for CBT. Perhaps more important, however, is the paucity of research on the effectiveness of IPT in patients with other forms of eating disorder, an exception being BED. As well as there being a need for more data to establish the effectiveness of IPT, research is also required to establish how IPT works. We are currently engaged in a research on IPT-induced mediators of change which we hope will be of help in identifying potential causal mechanisms through which the treatment operates (Murphy et al., 2009).
